# Liraglutide reduces lipid accumulation in human orbital fibroblasts in Graves’ orbitopathy

**DOI:** 10.3389/fendo.2026.1914378

**Published:** 2026-10-07

**Authors:** Chih-Wei Shih, Shyang-Rong Shih, Yi-Hsuan Wei, Chia-Hung Lin, Shu-Lang Liao, Ting-An Chou, I Chu, Hou-Jen Chen, Neng-Li Liao, Feng-Chiao Tsai

**Affiliations:** 1 Graduate Institute of Clinical Medicine, National Taiwan University College of Medicine, Taipei, Taiwan; 2 Department of Ophthalmology, Zhongxing Branch, Taipei City Hospital, Taipei, Taiwan; 3 Department of Ophthalmology, Good Will Clinic, Taipei, Taiwan; 4 Department of Internal Medicine, National Taiwan University Hospital, Taipei, Taiwan; 5 Department of Internal Medicine, National Taiwan University College of Medicine, Taipei, Taiwan; 6 Department of Ophthalmology, National Taiwan University Hospital, Taipei, Taiwan; 7 Department of Ophthalmology, National Taiwan University College of Medicine, Taipei, Taiwan; 8 Department of Pharmacology, National Taiwan University College of Medicine, Taipei, Taiwan

**Keywords:** glucagon-like peptide-1 receptor agonists, Graves’ orbitopathy, lipid accumulation, orbital fibroblasts, thyroid eye disease

## Abstract

Graves’ orbitopathy (GO) is characterized by proliferation and differentiation of orbital fibroblasts. Glucagon-like peptide-1 receptor agonists (GLP-1RAs) are widely used for metabolic disorders and have been reported to modulate cell proliferation and differentiation. The present study aimed to examine the effects of GLP-1RAs on key fibroblast-mediated pathogenic processes relevant to GO using human orbital fibroblasts. Orbital fibroblasts were isolated from patients with GO and non-GO controls. Cells were treated with the GLP-1RAs liraglutide (100 nM) or semaglutide (200 nM). Fibroblast proliferation was assessed by nuclear counting, lipid accumulation was evaluated by Oil Red O staining of oil droplets, and myofibroblastic differentiation was assessed by α-smooth muscle actin (α-SMA) immunostaining. Hyaluronan production was measured by ELISA. *GLP1R* mRNA expression was examined using reverse transcription quantitative PCR. Among the outcomes assessed, liraglutide significantly reduced lipid accumulation in GO-derived orbital fibroblasts, whereas semaglutide showed a nonsignificant trend in the same direction. Neither liraglutide nor semaglutide significantly altered lipid accumulation in non-GO fibroblasts. Importantly, neither GLP-1RA increased fibroblast proliferation, TGF-β–induced myofibroblastic differentiation, or IL-1β–induced hyaluronan production in GO or non-GO fibroblasts under the tested conditions. *GLP1R* mRNA was not reliably detected, and the underlying mechanism remains uncertain. Taken together, this *in vitro* study showed a significant reduction in lipid accumulation following liraglutide treatment in GO-derived orbital fibroblasts, while semaglutide showed a trend in the same direction that did not reach statistical significance. Neither agent enhanced fibroblast proliferation, myofibroblastic differentiation, or hyaluronan production under the tested conditions. These findings suggest a potentially favorable biological profile of liraglutide in GO and warrant further studies to clarify the underlying mechanisms and assess its clinical relevance, particularly in patients with metabolic comorbidities.

## 1 Introduction

Graves’ orbitopathy (GO), also referred to as thyroid orbitopathy or thyroid eye disease, is the most common orbital disorder and affects approximately 40% of patients with Graves’ disease (1). Manifestations include conjunctival injection, proptosis, eyelid retraction, restrictive extraocular myopathy, and, in severe cases, vision-threatening optic nerve compression (2). Despite advances in management, therapeutic options for GO remain limited and are often associated with incomplete responses or treatment-related adverse effects. For example, insulin-like growth factor-1 receptor (IGF-1R) inhibition demonstrates efficacy in moderate-to-severe GO; however, its use is limited by adverse effects, high cost, and disease relapse (3, 4). Conventional treatments, including glucocorticoids, mycophenolate sodium, rituximab, and radiotherapy, are also variably effective and may be poorly tolerated (5). Consequently, the development of alternative or adjunctive strategies targeting key pathogenic mechanisms in GO remains an unmet clinical need (2, 5–7).

GO is an autoimmune-mediated disorder characterized by infiltration of immune cells, including T cells, B cells, monocytes, and macrophages in orbital tissues. These cells interact with autoantibodies directed against the thyroid-stimulating hormone receptor and IGF-1R, as well as proinflammatory cytokines such as interleukins, tumor necrosis factor-α (TNF-α), transforming growth factor-β (TGF-β), and interferon-γ (8). Orbital fibroblasts play a pivotal role in GO pathophysiology. Upon activation, they proliferate, undergo adipogenic differentiation, and produce glycosaminoglycans, leading to tissue expansion and orbital remodeling (8). In addition, orbital fibroblasts may differentiate into myofibroblasts, contributing to fibrosis. Given their involvement in multiple pathogenic processes, orbital fibroblasts have been proposed as a potential therapeutic target in GO (7).

Glucagon-like peptide-1 (GLP-1) is an incretin hormone with pleiotropic biological effects mediated through both receptor-dependent and receptor-independent mechanisms. Beyond its metabolic actions, GLP-1 signaling has been implicated in the regulation of cellular proliferation, differentiation, and tissue remodeling in multiple mesenchymal cell types (9–12). Experimental studies further suggest a role for GLP-1–related signaling in adipocyte differentiation and lipogenesis in human stem cells, 3T3-L1 cells, and rodent adipocytes (13–20). These findings suggest that GLP-1–related pathways may influence central pathogenic features of GO, including adipogenic and fibrotic processes; however, the effects of GLP-1 receptor agonists (GLP-1RAs) on human orbital fibroblasts have not been systematically examined.

GLP-1RAs are widely prescribed for type 2 diabetes mellitus and obesity and exert biological effects beyond metabolic control, including anti-inflammatory and immunomodulatory actions (12, 21–23). Emerging clinical data suggest ocular associations with GLP-1RAs, with reports describing both protective and adverse outcomes across retinal and optic nerve disorders (24–26). Given the high prevalence of metabolic comorbidities in Graves’ disease and their association with more severe GO (27, 28), it is clinically relevant to clarify whether GLP-1RAs influence disease-relevant orbital fibroblast functions.

In this study, we investigated the effects of GLP-1RAs on human orbital fibroblasts, focusing on proliferation, lipid accumulation, myofibroblastic differentiation, and hyaluronan production, to assess their potential biological impact in the context of GO.

## 2 Materials and methods

### 2.1 Cell culture

Orbital fibroblasts were obtained from patients with GO undergoing orbital decompression surgery and from non-GO subjects undergoing plastic surgery at National Taiwan University Hospital. A total of ten patients with GO (GO group) and ten healthy controls (non-GO group) participated in the study. **Table 1** summarizes their clinical characteristics. All GO patients were euthyroid and demonstrated low disease activity. Orbital tissues were collected during surgery, and orbital fibroblasts were isolated as previously described (29). Cells were cultured in Dulbecco’s Modified Eagle’s Medium/Ham’s F-12 (DMEM/F-12; Gibco, Thermo Fisher Scientific, Waltham, MA, USA) supplemented with 20% fetal bovine serum (FBS) and 1% penicillin–streptomycin (P/S). Cells between passages 3 and 5 were used for all experiments. For pharmacological treatment experiments, GLP-1RAs, including liraglutide (MedChemExpress, Monmouth Junction, NJ, USA; Cat. No. HY-P0014; purity > 99%) and semaglutide (MedChemExpress; Cat. No. HY-114118; purity > 99%) were used. Liraglutide was dissolved in H_2_O and adjusted to pH 3 with 1 M HCl, whereas semaglutide was dissolved in 0.5 M NH_4_OH. Stock solutions were aliquoted and stored at −30°C until use. Working solutions were prepared by dilution in culture medium to final concentrations of 100 nM liraglutide and 200 nM semaglutide. Separate solvent-matched vehicle controls were not included. The concentrations of liraglutide and semaglutide were selected based on prior *in vitro* studies demonstrating effective GLP-1 receptor (GLP-1R) activation in mesenchymal and stromal cell models using nanomolar concentrations (30, 31). These concentrations were additionally validated in pilot experiments in orbital fibroblasts to ensure cellular viability under the experimental conditions used.

**Table 1:** Characteristics of study subjects.

Variable	GO	Non-GO
Age (years)*	42 (31–49)	56 (48–67)
Sex (male:female)^†^	2:8	4:6
fT4 (ng/dL)	1.08 (0.92–1.27)	—
TSH (mU/L)	1.02 (0.30–1.80)	—
TBII (%)	15.5 (4.0–26.9)	—
Clinical activity score	1.5 (1–2)	—

Data are presented as median (interquartile range).

fT4, free thyroxine; GO, Graves’ orbitopathy; Non-GO, subjects without Graves’ orbitopathy; TBII, thyrotropin-binding inhibitory immunoglobulin; TSH, thyroid-stimulating hormone.

*Age differed significantly between GO and non-GO groups (Wilcoxon–Mann–Whitney test, p < 0.05).

^†^Sex distribution did not differ significantly between groups (Fisher’s exact test, p = 0.63).

This study was approved by the Research Ethics Committee of National Taiwan University Hospital (NTUH-REC No.: 201412026RINB) and was conducted in accordance with the Declaration of Helsinki. Written informed consent was obtained from all participants prior to tissue collection.

### 2.2 Proliferation assay

Orbital fibroblasts were seeded at 3,000 cells per well in collagen-coated 96-well plates and allowed to adhere overnight. The culture medium was then replaced with DMEM/F-12 containing 0.1% FBS and 1% P/S, with or without GLP-1RAs (liraglutide: 100 nM or semaglutide: 200 nM) for 72 hours. Nuclei were stained with 4',6-diamidino-2-phenylindole (DAPI), and cell numbers were determined by manual counting in 9 randomly selected fields under a fluorescence microscope (Eclipse Ti; Nikon, Tokyo, Japan). Experiments were performed in technical triplicates for each donor and treatment condition.

### 2.3 Lipid accumulation assay

Cells were stained with Oil Red O (ORO) to detect oil droplets. Fibroblasts were plated at a density of 5,000 cells per well in collagen-coated 96-well plates and allowed to attach for 24 hours. The cultures were then maintained in DMEM/F-12 containing 20% FBS and 1% P/S, either in the absence or presence of GLP-1RAs (liraglutide: 100 nM or semaglutide: 200 nM) for 12 days, with medium renewed every two days. At the end of the 12-day culture period, cells were fixed with 4% paraformaldehyde and stained with ORO to visualize lipid accumulation and DAPI to mark nuclei. The fluorescence intensity ratio of ORO to DAPI-stained nuclei was calculated to quantitatively evaluate lipid accumulation of fibroblasts under a fluorescence microscope (Eclipse Ti; Nikon, Tokyo, Japan).

### 2.4 Myofibroblastic differentiation assay

Fibroblasts were seeded at 3,000 cells per well and allowed to adhere overnight. The cultures were then switched to DMEM/F-12 supplemented with 0.1% FBS, 1% P/S, and TGF-β (10 ng/mL) for 48 hours, with or without GLP-1RAs (liraglutide: 100 nM or semaglutide: 200 nM). Cells were fixed on ice for 5 minutes using acetone/methanol (1:1) and subsequently blocked in 5% bovine serum albumin (BSA) in phosphate-buffered saline (PBS) for 1 hour. To evaluate myofibroblastic differentiation, α-smooth muscle actin (α-SMA) was detected by incubating the cells overnight at 4°C with a primary antibody (1:100; ab5694, Abcam, Cambridge, UK) diluted in PBS containing 1% BSA. The next day, cells were exposed to Alexa Fluor 594-conjugated secondary antibodies (1:300; Invitrogen, Thermo Fisher Scientific, Waltham, MA, USA) for 1 hour. Nuclear counterstaining was performed with 10 μg/mL DAPI in PBS for 15 minutes. Fluorescence signals from α-SMA and nuclei were visualized with a fluorescence microscope (Eclipse Ti; Nikon, Tokyo, Japan), and the ratio of α-SMA intensity to DAPI-stained nuclei was calculated to quantify myofibroblastic differentiation. Experiments were performed in technical triplicates for each donor and treatment condition.

### 2.5 Hyaluronan quantification

Orbital fibroblasts were plated in collagen-coated 96-well plates at a density of 5,000 cells per well and incubated overnight to allow cell attachment. The medium was then replaced with DMEM/F-12 supplemented with 0.1% FBS, 1% P/S, and interleukin-1β (IL-1β; 10 ng/mL), with or without GLP-1RAs (liraglutide: 100 nM or semaglutide: 200 nM). After 24 hours of incubation, culture supernatants were harvested, and hyaluronan concentrations were determined using a commercial enzyme-linked immunosorbent assay (ELISA) kit (Quantikine Hyaluronan ELISA; R&D Systems, Minneapolis, MN, USA) according to the manufacturer’s protocol.

### 2.6 GLP-1R expression

*GLP1R* Gene expression was assessed by reverse transcription quantitative PCR (RT-qPCR). Total RNA was extracted using RNA Isolater Total RNA Extraction Reagent (Vazyme, China). Reverse transcription was performed using HiScript III RT SuperMix for qPCR (+gDNA wiper; Vazyme, China), which includes a genomic DNA removal step prior to cDNA synthesis, according to the manufacturer’s instructions (42°C for 2 min for gDNA digestion, 37°C for 15 min for reverse transcription, and 85°C for 5 s for enzyme inactivation), using 1,000 ng total RNA per reaction. Quantitative PCR was performed using Taq Pro Universal SYBR qPCR Master Mix (Vazyme, China) in 10 μL reactions on a StepOnePlus Real-Time PCR System (Applied Biosystems, USA). *GLP1R* expression was assessed using multiple independent SYBR Green primer sets spanning different regions of the transcript, with *HPRT1* used as an internal reference. Each reaction contained 20 ng cDNA and was run in technical triplicate with no-template and no–reverse transcription controls. Cycle threshold (Ct) values ≥ 35 were considered below the reliable detection range. Assay performance for *GLP1R* was verified using RNA from Jurkat cells (clone E6-1) as a positive control. Primer sequences used for RT-qPCR are listed in **Supplementary Table S1**.

GLP-1R protein expression was additionally assessed by Western blotting using orbital fibroblasts from two GO donors. Total cellular protein was extracted using radioimmunoprecipitation assay (RIPA) lysis buffer supplemented with a protease inhibitor cocktail, and equal amounts of protein (20 μg) were separated by sodium dodecyl sulfate–polyacrylamide gel electrophoresis (SDS-PAGE) and transferred to polyvinylidene difluoride (PVDF) membranes. Membranes were incubated with a rabbit monoclonal anti-GLP-1R antibody (Abcam, Cambridge, UK; ab218532, clone EPR21819; 1:1000) at 4°C overnight, followed by a horseradish peroxidase-conjugated secondary antibody. GAPDH was used as a loading control. Each donor was examined in technical triplicate.

### 2.7 Exploratory assessment of adipogenic transcriptional markers

To assess adipogenic molecular changes under the 12-day lipid accumulation condition (DMEM/F-12 with 20% FBS), orbital fibroblasts from two GO donors were evaluated at Day 0 and Day 12 without GLP-1RA treatment. Total RNA extraction and RT-qPCR were performed as described above. Expression of *PPARG* and *CEBPA* was assessed using gene-specific SYBR Green primers, with *HPRT1* as the reference gene. Reactions were performed in technical triplicate and averaged for each donor. Relative expression was calculated using the 2^−ΔΔCt^ method, with the corresponding Day 0 sample used as the calibrator and set to 1. Primer sequences are listed in **Supplementary Table S1**.

### 2.8 Exploratory adipogenic induction and IGF-1R inhibition experiments

To further characterize lipid accumulation under different culture conditions, orbital fibroblasts from two GO donors were cultured for 12 days in DMEM/F-12 containing either 20% FBS, 10% FBS, or 10% FBS supplemented with an adipogenic induction cocktail consisting of 0.5 mM 3-isobutyl-1-methylxanthine (IBMX), 1 μM dexamethasone, and 0.1 μM insulin. The culture medium was renewed every two days. At the end of the 12-day culture period, cells were stained with ORO and DAPI. The fluorescence intensity ratio of ORO to DAPI-stained nuclei was calculated to quantitatively evaluate lipid accumulation of fibroblasts under a fluorescence microscope (Eclipse Ti; Nikon, Tokyo, Japan). Experiments were performed with six technical replicates for each donor and treatment condition.

To explore the potential involvement of IGF-1R-related signaling, additional exploratory experiments were performed in the same two GO donors under the adipogenic induction condition described above (10% FBS supplemented with IBMX, dexamethasone, and insulin). Cells were cultured for 12 days with picropodophyllin (PPP; 100 nM; MedChemExpress, Monmouth Junction, NJ, USA; Cat. No. HY-15494; purity > 99%), an IGF-1R inhibitor, either alone or in combination with liraglutide (100 nM) or semaglutide (200 nM). PPP was dissolved in DMSO, and stock solutions were aliquoted and stored at −30°C until use. Working solutions were prepared by dilution in culture medium. The culture medium and treatments were renewed every two days. At the end of the treatment period, cells were stained with ORO and DAPI, and DAPI-normalized ORO signal was quantified as described above. Experiments were performed with six technical replicates for each donor and treatment condition.

### 2.9 Statistics

All statistical analyses were performed using Stata/SE version 14.0 for Windows (StataCorp, College Station, TX, USA). For comparison of baseline characteristics between GO and non-GO groups, continuous variables are presented as median (interquartile range) and were compared using the Wilcoxon–Mann–Whitney test. Categorical variables were compared using Fisher’s exact test. For the proliferation, ORO, and myofibroblastic differentiation assays, measurements were performed in technical triplicate for each donor and condition. Technical replicates were averaged to obtain one value per donor per condition. For hyaluronan quantification, one ELISA measurement was obtained per donor per condition. For graphical presentation, donor-level values were normalized to the mean untreated control value within each group, with the control mean set to 1. Differences following treatment with GLP-1RAs were evaluated using paired Student’s t tests for donor-matched samples. Spearman rank correlation analyses were performed to assess associations between donor age and liraglutide or semaglutide responses. A p value < 0.05 was considered statistically significant. Treatment response was defined as the ratio of treated to control DAPI-normalized ORO signal. The exploratory adipogenic induction and IGF-1R inhibition experiments were performed in two GO donors, with six technical replicates per donor and condition. Results are presented descriptively as mean ± SD of the technical replicates for each donor. Because only two biological donors were examined, no inferential statistical testing was performed.

## 3 Results

### 3.1 GLP-1RAs did not significantly affect the proliferation of orbital fibroblasts

The number of orbital fibroblasts was quantified by DAPI staining, and no significant change was detected following treatment with liraglutide (100 nM) or semaglutide (200 nM) in either the GO group (**Figures 1A, B**) or the non-GO group (**Figures 1C, D**).

**Figure 1:**
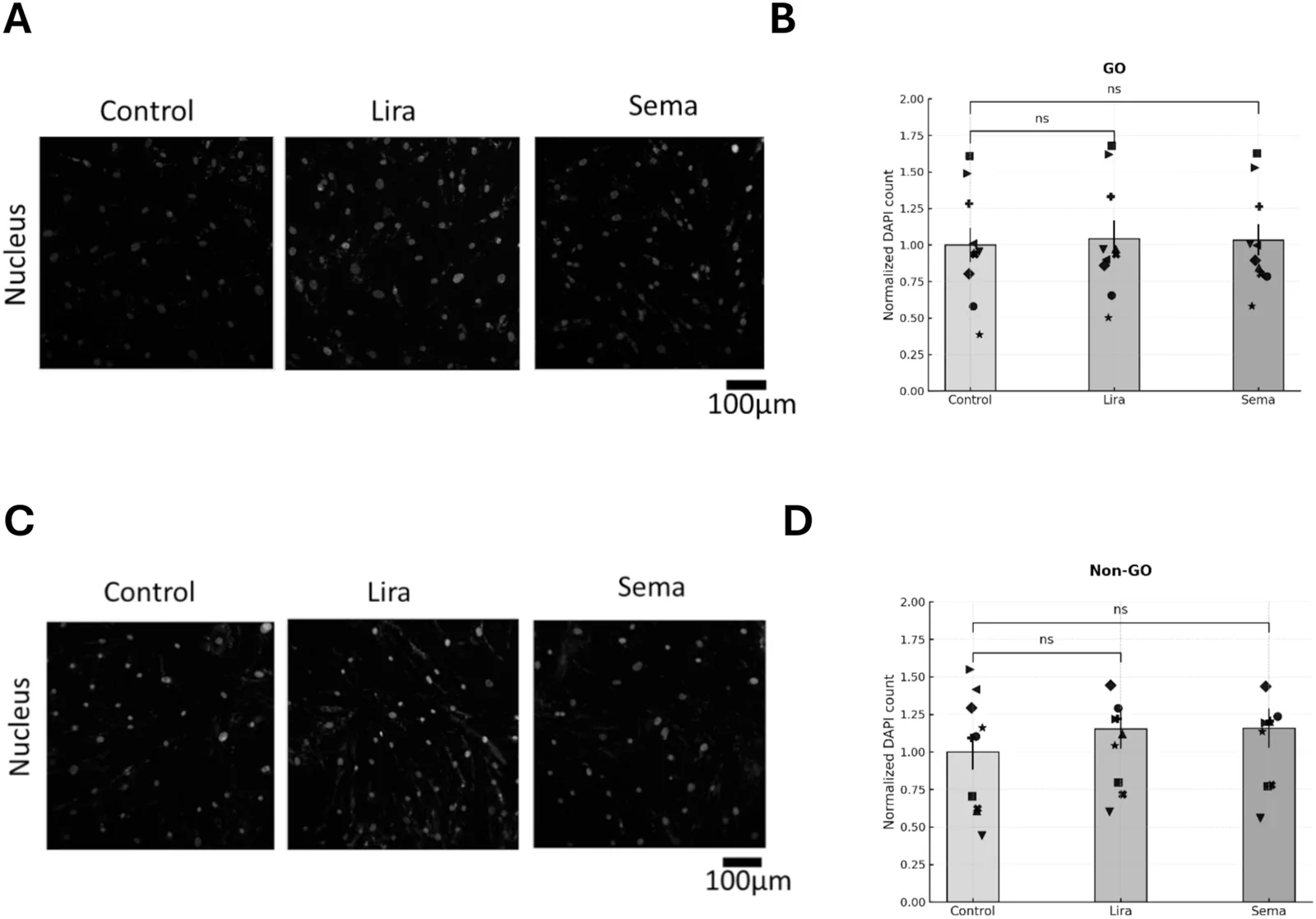
GLP-1 receptor agonists do not affect proliferation of orbital fibroblasts from GO or non-GO subjects. Orbital fibroblasts derived from GO (n = 10 donors) and non-GO (n = 10 donors) subjects were cultured in the presence or absence of liraglutide (100 nM) or semaglutide (200 nM) for 72 hours. Nuclei were stained with 4′,6-diamidino-2-phenylindole (DAPI) to quantify cell numbers. **(A)** Representative DAPI staining images of GO orbital fibroblasts. **(B)** Quantification of DAPI-positive nuclei in GO orbital fibroblasts. **(C)** Representative DAPI staining images of non-GO orbital fibroblasts. **(D)** Quantification of DAPI-positive nuclei in non-GO orbital fibroblasts. Technical triplicates were averaged to obtain one donor-level value per condition and normalized to the mean untreated control value within each group, with the control mean set to 1. Treatment effects were assessed using paired Student’s t tests. Data are presented as mean ± standard error of the mean (SEM) from biological donors. ns, not significant.

### 3.2 Liraglutide reduced lipid accumulation in GO-derived orbital fibroblasts

Lipid accumulation was assessed by ORO staining in orbital fibroblasts. In GO-derived fibroblasts, liraglutide (100 nM) reduced DAPI-normalized ORO signal compared with untreated controls (p < 0.05), with a mean donor-level treatment ratio (liraglutide/control) of 0.886, corresponding to an average reduction of approximately 11.4%. Semaglutide (200 nM) showed a nonsignificant trend in the same direction, with a mean donor-level treatment ratio of 0.940. In the non-GO group, neither liraglutide nor semaglutide significantly altered DAPI-normalized ORO signal compared with untreated controls (**Figures 2C, D**). The corresponding donor-level DAPI-normalized ORO signal values are provided in **Supplementary Table S2**. Because age differed significantly between the GO and non-GO groups (**Table 1**), exploratory within-group Spearman correlation analyses were performed. No significant associations were observed between age and treatment response for either liraglutide (GO: ρ = 0.220, p = 0.542; non-GO: ρ = 0.384, p = 0.273) or semaglutide (GO: ρ = 0.079, p = 0.828; non-GO: ρ = −0.433, p = 0.211).

**Figure 2:**
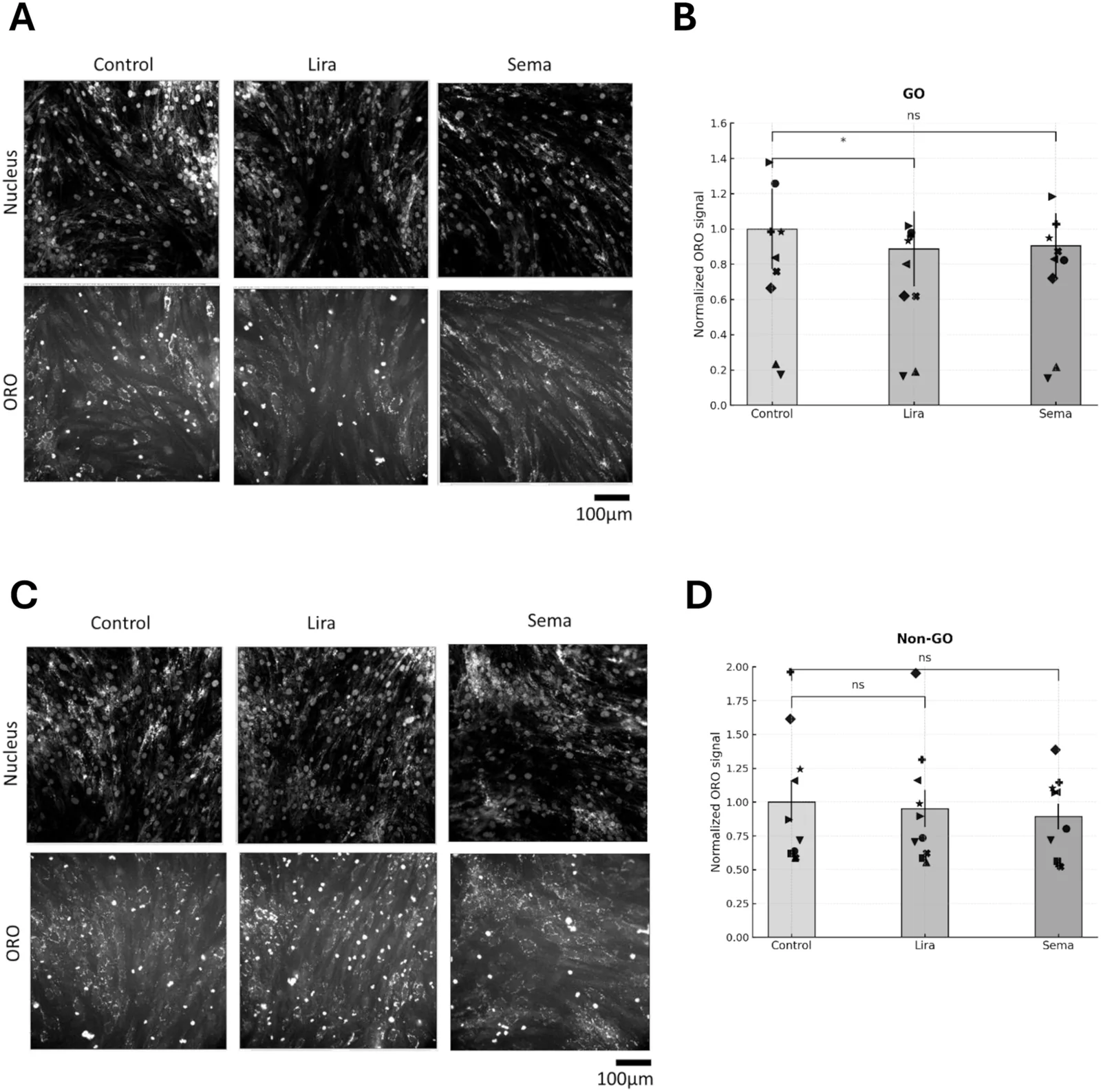
Effects of GLP-1 receptor agonists on lipid accumulation in GO and non-GO orbital fibroblasts. Orbital fibroblasts derived from GO (n = 10 donors) and non-GO (n = 10 donors) subjects were cultured for 12 days in the presence or absence of liraglutide (100 nM) or semaglutide (200 nM). Lipid accumulation was assessed by Oil Red O (ORO) staining. **(A)** Representative ORO staining images of GO orbital fibroblasts. **(B)** Quantification of normalized ORO staining intensity in GO orbital fibroblasts. **(C)** Representative ORO staining images of non-GO orbital fibroblasts. **(D)** Quantification of DAPI-normalized ORO staining intensity in non-GO orbital fibroblasts. ORO fluorescence intensity was normalized to DAPI-stained nuclei. Technical triplicates were averaged to obtain one donor-level DAPI-normalized ORO signal per condition. Donor-level values were then normalized to the mean untreated control value within each group, with the control mean set to 1. Treatment effects were assessed using paired Student’s t tests. Data are presented as mean ± SEM from biological donors. ns, not significant; *p < 0.05.

To further characterize the 12-day culture condition, exploratory RT-qPCR analysis was performed in orbital fibroblasts from two GO donors. Under the tested culture conditions without GLP-1RA treatment, expression of *PPARG* and *CEBPA* increased from Day 0 to Day 12 in both donors (**Supplementary Figure S1**).

In an additional exploratory adipogenic induction experiment using two GO donors, DAPI-normalized ORO signal was compared among cultures containing 20% FBS, 10% FBS, and 10% FBS supplemented with an adipogenic induction cocktail containing insulin, dexamethasone, and IBMX. Addition of the induction cocktail to 10% FBS did not further increase the DAPI-normalized ORO signal compared with 10% FBS alone in either donor (**Supplementary Figures S3A, B**). In exploratory IGF-1R inhibition experiments using the same two GO donors under adipogenic induction conditions, neither liraglutide nor semaglutide further reduced the DAPI-normalized ORO signal in the presence of PPP compared with PPP treatment alone (**Supplementary Figures S3C, D**).

### 3.3 GLP-1RAs had no significant effect on myofibroblastic differentiation of orbital fibroblasts

α-SMA staining was used to evaluate myofibroblastic differentiation following TGF-β stimulation, and no significant change was detected following treatment with liraglutide (100 nM) or semaglutide (200 nM) in either the GO group (**Figures 3A, B**) or the non-GO group (**Figures 3C, D**).

**Figure 3:**
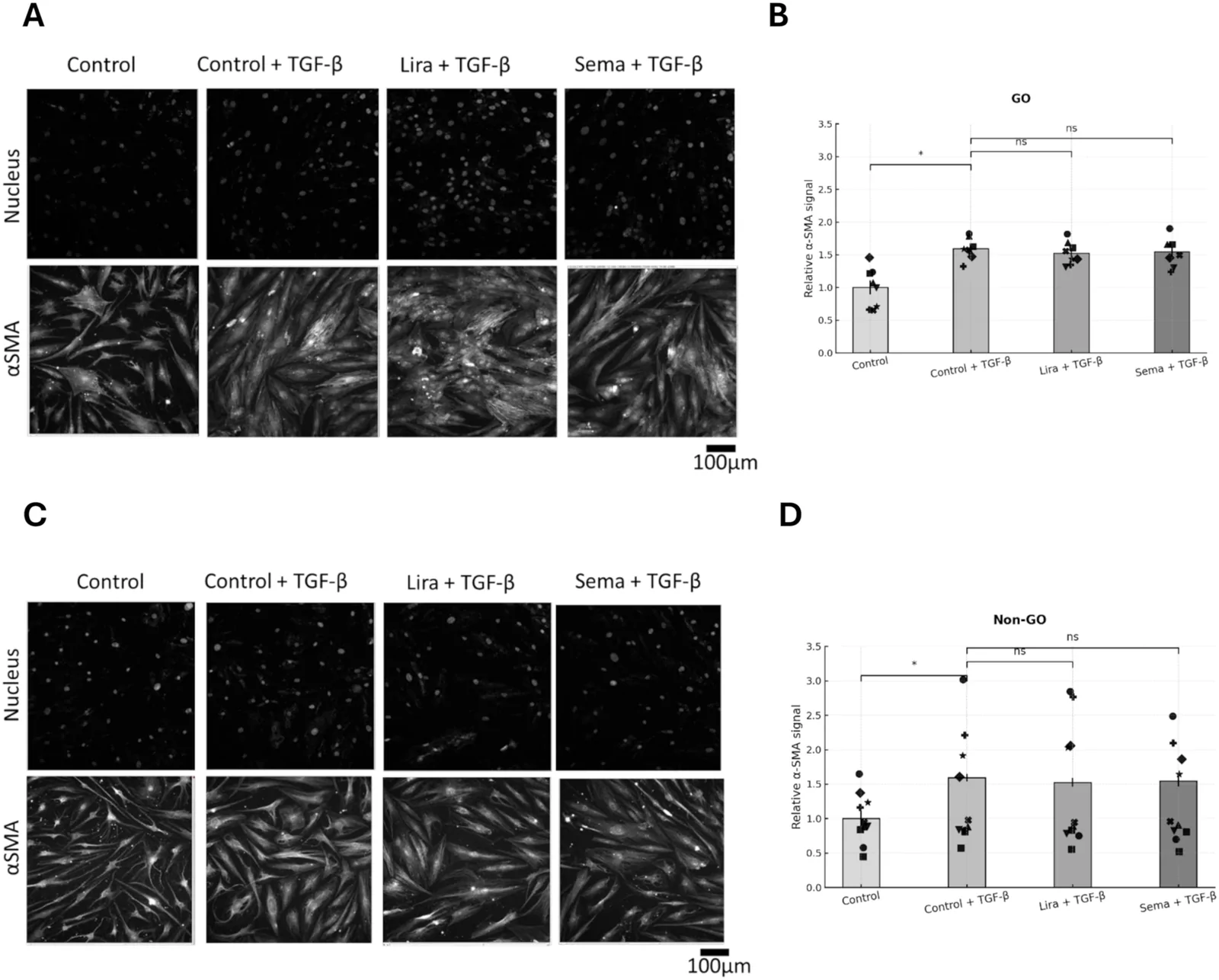
Effects of GLP-1 receptor agonists on TGF-β–induced myofibroblastic differentiation in GO and non-GO orbital fibroblasts. Orbital fibroblasts derived from GO (n = 10 donors) and non-GO (n = 10 donors) subjects were stimulated with transforming growth factor-β (TGF-β, 10 ng/mL) in the presence or absence of liraglutide (100 nM) or semaglutide (200 nM) for 48 hours. Myofibroblastic differentiation was assessed by α-smooth muscle actin (α-SMA) immunofluorescence staining. **(A)** Representative α-SMA staining images of GO orbital fibroblasts. **(B)** Quantification of normalized α-SMA fluorescence intensity in GO orbital fibroblasts. **(C)** Representative α-SMA staining images of non-GO orbital fibroblasts. **(D)** Quantification of normalized α-SMA fluorescence intensity in non-GO orbital fibroblasts. α-SMA fluorescence intensity was normalized to DAPI-stained nuclei. Technical triplicates were averaged to obtain one donor-level value per condition and normalized to the mean untreated control value within each group, with the control mean set to 1. Treatment effects were assessed using paired Student’s t tests. Data are presented as mean ± SEM from biological donors. ns, not significant. *p < 0.05.

### 3.4 GLP-1RAs did not significantly affect hyaluronan production

GLP-1RAs were evaluated for their effects on IL-1β–induced hyaluronan production in orbital fibroblasts. In both GO and non-GO groups, IL-1β stimulation markedly increased hyaluronan levels compared with controls, while treatment with liraglutide (100 nM) or semaglutide (200 nM) did not significantly attenuate this induction (**Figure 4**).

**Figure 4:**
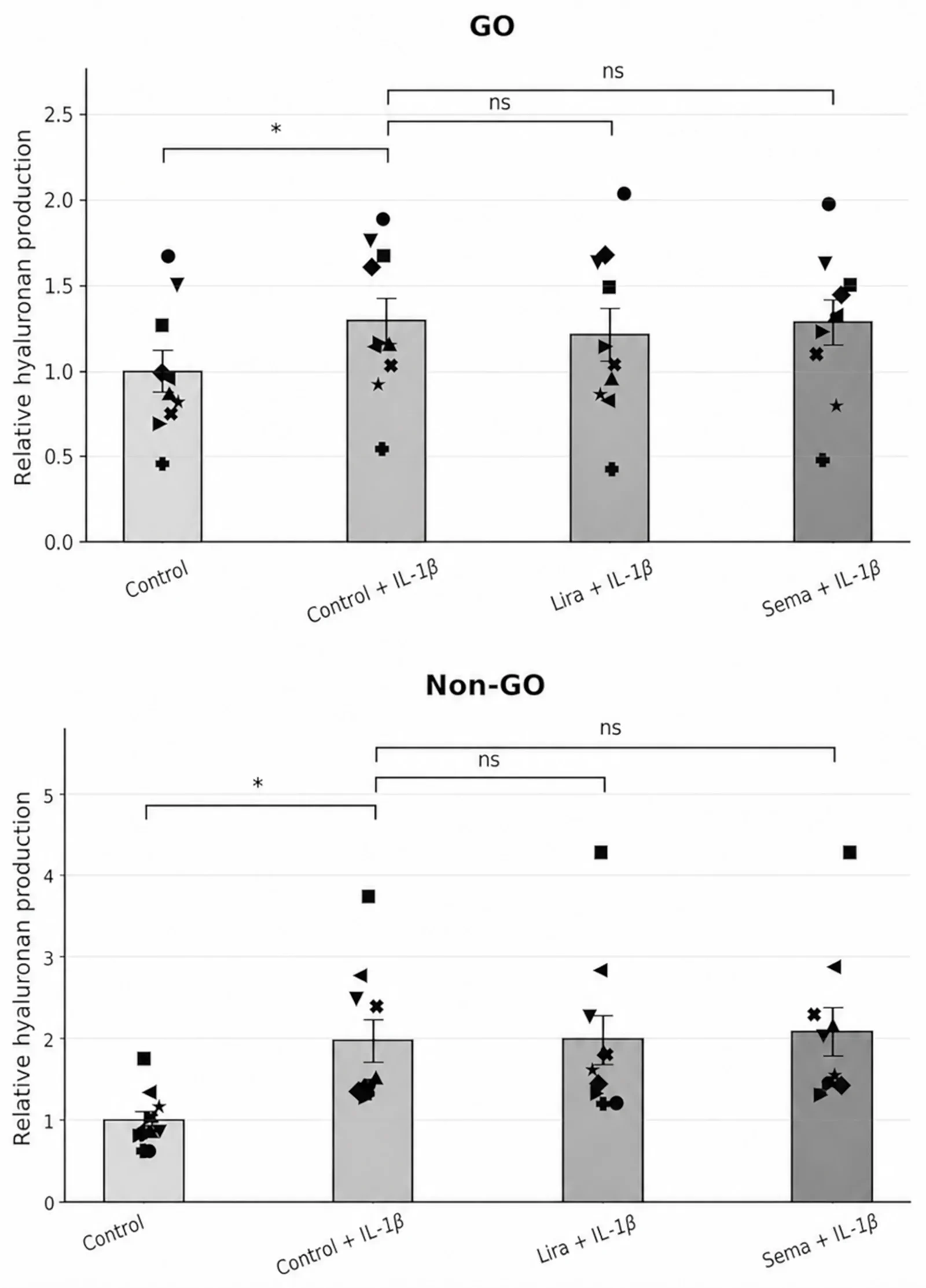
Effects of GLP-1 receptor agonists on IL-1β–induced hyaluronan production in orbital fibroblasts. Orbital fibroblasts derived from GO (n = 10 donors) and non-GO (n = 10 donors) subjects were stimulated with interleukin-1β (IL-1β, 10 ng/mL) in the presence or absence of liraglutide (100 nM) or semaglutide (200 nM) for 24 hours. Hyaluronan levels in culture supernatants were quantified by ELISA. For graphical presentation, donor-level values were normalized to the mean untreated control value within each group, with the control mean set to 1. Treatment effects were assessed using paired Student’s t tests. Data are presented as mean ± SEM from biological donors. ns, not significant; *p < 0.05.

### 3.5 GLP-1R expression was not reliably detected in orbital fibroblasts

*GLP1R* mRNA was not reliably detected in orbital fibroblasts from either the GO or non-GO groups, with Ct values either undetermined or ≥35 across multiple primer sets (**Supplementary Table S1**). In an additional exploratory protein-level assessment, Western blotting of orbital fibroblasts from two GO donors did not reveal a clear specific GLP-1R band (**Supplementary Figure S2**).

## 4 Discussion

In this study, liraglutide significantly reduced lipid accumulation in orbital fibroblasts derived from patients with GO, whereas semaglutide showed a nonsignificant trend in the same direction. Neither agent significantly affected fibroblast proliferation, TGF-β–induced myofibroblastic differentiation, or IL-1β–induced hyaluronan production under the tested conditions. Taken together, these findings suggest that liraglutide reduced lipid accumulation without exacerbating the other fibroblast-mediated pathogenic responses examined in this study.

The influence of GLP-1 and GLP-1RAs on adipogenesis has been reported in multiple experimental models. In human adipose-derived stem cells, liraglutide inhibits adipogenic differentiation and counteracts insulin-driven lipid storage (14, 19). In human bone marrow–derived mesenchymal stem cells, GLP-1 enhances cellular proliferation while inhibiting adipocyte differentiation (13). In 3T3-L1 adipocytes, liraglutide suppresses fatty acid synthase, a key enzyme involved in *de novo* lipogenesis (16). Animal studies further demonstrate that GLP-1 and its analogs reduce hepatic lipid content and downregulate lipogenic gene expression in high-fat–fed mice (17). In contrast, other studies have reported pro-adipogenic effects of GLP-1 in 3T3-L1 preadipocytes (15, 16, 32), suggesting that the effects of GLP-1-related signaling on adipogenesis may depend on cell type and differentiation stage.

In our study, RT-qPCR analysis in two GO donors showed increased *PPARG* and *CEBPA* expression from Day 0 to Day 12, consistent with partial activation of an adipogenic program under the tested culture conditions. However, because only two donors were examined, these findings were considered exploratory. In the same donors, addition of an adipogenic induction cocktail did not further increase DAPI-normalized ORO signal, suggesting that substantial lipid accumulation can occur under these culture conditions. However, these findings do not establish complete adipogenic differentiation. Accordingly, the observed reduction in ORO staining is more appropriately interpreted as reduced lipid accumulation, and whether this reflects inhibition of adipogenic differentiation or altered lipid storage or metabolism remains to be determined.

In the present study, *GLP1R* mRNA was not reliably detected in GO and non-GO orbital fibroblasts, and Western blotting did not reveal a clear specific GLP-1R signal in two GO donors. Although low-level receptor expression cannot be excluded, these findings raise the possibility that the observed effects of liraglutide may not require robust classical GLP-1R signaling. Previous studies have shown that GLP-1–related effects may occur through canonical GLP-1R-dependent pathways involving PKA, MAPK, Akt, and PKC signaling (13, 15, 16). In contrast, GLP-1–related effects may also occur through noncanonical mechanisms, including signaling through alternative receptors such as IGF-1R (18, 33–35). In exploratory experiments using two GO donors, liraglutide and semaglutide did not further reduce DAPI-normalized ORO signal in the presence of PPP, consistent with possible involvement of IGF-1R-related signaling. However, because these experiments used different culture conditions and only two donors, the findings remain exploratory.

Recent clinical and observational studies have reported mixed ocular outcomes associated with GLP-1RA use, including associations related to diabetic retinopathy progression, nonarteritic anterior ischemic optic neuropathy, and neovascular age-related macular degeneration in selected patient populations, while other studies suggest potential protective associations in chronic retinal diseases (24–26). These observations highlight the complexity of GLP-1–related signaling in ocular tissues and underscore the importance of evaluating tissue- and pathway-specific effects of GLP-1RAs. In this context, our findings demonstrate that GLP-1RAs do not enhance proliferation, fibrotic differentiation, or hyaluronan production in GO-derived fibroblasts, while liraglutide was associated with a significant reduction in lipid accumulation and semaglutide showed a nonsignificant trend in the same direction. Although these *in vitro* results cannot be extrapolated to overall ocular safety or clinical outcomes, they suggest that GLP-1RAs do not exacerbate key fibroblast-mediated pathogenic processes relevant to GO.

Liraglutide reduced lipid accumulation in orbital fibroblasts derived from GO patients, whereas no significant change was observed in non-GO fibroblasts. This pattern may reflect intrinsic biological differences in GO orbital fibroblasts, although differences in donor characteristics and tissue sampling may also have contributed. Previous studies have shown that orbital fibroblasts from patients with GO are predisposed to adipogenic differentiation, associated with upregulated PPARγ and C/EBPα signaling, increased IGF-1R activity, and heightened oxidative stress (36, 37). However, the GO donors were significantly younger than the non-GO donors, with median ages of 42 and 56 years, respectively, and the tissues were obtained in different surgical contexts: orbital decompression in the GO group and plastic surgery in the non-GO group. Age, surgical indication, anatomical sampling, and other unmeasured donor characteristics may therefore have influenced fibroblast phenotype, lipid metabolism, and responsiveness to GLP-1RAs. In exploratory analyses, age was not significantly associated with the response to either liraglutide or semaglutide within either group. However, the small sample size and limited age distribution within each group do not exclude residual age-related confounding or explain the between-group differences. Because all GO donors were euthyroid, had low disease activity, and underwent orbital decompression, the findings may primarily reflect chronic, inactive, or adipose-predominant GO and should not be extrapolated to active disease. Larger age-matched studies across different disease activities are needed to determine whether GLP-1RA responsiveness is associated with GO status.

Prior studies in non-orbital tissues have suggested that GLP-1RAs may attenuate fibrotic responses in organs such as the liver, kidney, and heart (38–40). In contrast, our data indicate that neither liraglutide nor semaglutide significantly altered TGF-β–induced myofibroblastic differentiation or IL-1β–induced hyaluronan production in orbital fibroblasts. The absence of comparable antifibrotic effects in orbital fibroblasts may reflect tissue-specific regulatory mechanisms, differences in receptor expression, or distinct inflammatory microenvironmental requirements.

Previous attempts to therapeutically target fibroblast-mediated tissue remodeling in GO have underscored the challenge of selectively modulating disease pathways. For example, pharmacologic inhibition of fibroblast growth factor signaling suppresses adipogenesis in orbital fibroblasts but concurrently enhances myofibroblastic differentiation, thereby limiting therapeutic applicability (29). In contrast, the reduction in lipid accumulation associated with liraglutide in the present study was not accompanied by exacerbation of fibrotic responses, highlighting its potential relevance for fat-dominant GO. Moreover, GLP-1RAs are already clinically approved and possess well-characterized safety profiles, providing a practical opportunity for therapeutic repurposing in patients with GO and coexisting metabolic disorders (41–43). In this context, metabolic status may also influence orbital fibroblast behavior and treatment response. Clinically, glycated hemoglobin (HbA1c) reflects a continuous spectrum of metabolic stress and microvascular vulnerability, with changes detectable even below the diagnostic threshold for diabetes (44, 45). Experimental studies further suggest that hyperglycemia can either enhance or impair adipogenesis depending on the duration of exposure and cellular context (46, 47). Future studies are needed to clarify the relationship between glycemic status, orbital fibroblast lipid accumulation, and responsiveness to GLP-1RAs.

Several limitations of this study should be acknowledged. The relatively small sample size limited statistical power and precluded subgroup analyses based on disease severity, treatment history, or clinical activity. Given the modest magnitude of the observed liraglutide-associated reduction and the relatively high *in vitro* drug concentrations used (48), the biological and translational significance of these findings should be interpreted cautiously. Because separate solvent-matched vehicle controls were not included for either the GLP-1RA treatments or the PPP experiments, potential vehicle-related effects cannot be entirely excluded. Future studies should examine dose-response relationships and different treatment durations at clinically relevant exposures, while incorporating appropriate solvent-matched vehicle controls. Mechanistic assessment was limited to GLP-1R expression and IGF-1R inhibition experiments in a small number of donors. These findings therefore remain exploratory, and the pathways mediating the observed effects require further investigation.

This study provides preliminary evidence that GLP-1RAs did not exacerbate the examined fibroblast-mediated pathogenic responses, while liraglutide reduced lipid accumulation under the present *in vitro* conditions. Given the widespread clinical use of GLP-1RAs for type 2 diabetes and obesity, understanding their effects on GO is increasingly relevant, particularly in patients with coexisting metabolic disorders. Further clinical and translational studies are needed to determine whether these *in vitro* observations are reproducible *in vivo* and relevant to patients with GO.

## Data Availability

The raw data supporting the conclusions of this article will be made available by the authors, without undue reservation.
