# Supplementary material for: Liraglutide reduces lipid accumulation in human orbital fibroblasts in Graves’ orbitopathy
Source: Front Endocrinol (Lausanne). 2026 Oct 7;17:1914378. doi: 10.3389/fendo.2026.1914378 (PMC13682971; doi:10.3389/fendo.2026.1914378)
Supplement: Supplementary file 1 [file Table1.docx]

**Supplementary Table S1. Primer sequences used for quantitative PCR**

| **Gene** | **Primer set** | **Forward primer (5′–3′)** | **Reverse primer (5′–3′)** |
| --- | --- | --- | --- |
| *GLP1R* | Set 1 | CTACGCACTCTCCTTCTCTGCT | CGGACAATGCTCGCAGGATGAA |
| *GLP1R* | Set 2 | GGCTGATGGTGGCCATATTATAC | TTCATGCTGCTGTCCCTCTG |
| *GLP1R* | Set 3 | CCTCCTCGGCTTCAGACAC | GGGCGGCTGTGCTATACATC |
| *PPARG* | — | TCTGGATTTCACTATGGAGTTCATG | TCCGACAGTTAAGATCACATCTGTC |
| *CEBPA* | — | CGGTGGCTTCTCCCTGC | GACAAGGCACGATTTGCTCC |
| *HPRT1* | — | TCATTATGCTGAGGATTTGGAAAG | GCCTCCCATCTCCTTCATCA |

Note. Multiple primer sets targeting different regions of *GLP1R* were tested. *GLP1R* primer set 1 was provided by Topgen Biotech (Taiwan), whereas *GLP1R* primer sets 2 and 3 were designed in-house using Primer-BLAST. Primers for *PPARG*, *CEBPA*, and *HPRT1* were provided by Topgen Biotech (Taiwan). *HPRT1* was used as the reference gene for normalization.
