# Supplementary material for: Liraglutide reduces lipid accumulation in human orbital fibroblasts in Graves’ orbitopathy
Source: Front Endocrinol (Lausanne). 2026 Oct 7;17:1914378. doi: 10.3389/fendo.2026.1914378 (PMC13682971; doi:10.3389/fendo.2026.1914378)
Supplement: Supplementary file 2 [file Table2.docx]

Supplementary Table S2. Donor-level DAPI-normalized ORO values in GO and non-GO orbital fibroblasts

| **Group** | **Donor** | **Control** | **Liraglutide (100 nM)** | **Semaglutide (200 nM)** |
| --- | --- | --- | --- | --- |
| GO | 1 | 369.80 | 288.01 | 242.01 |
| GO | 2 | 404.82 | 299.01 | 347.72 |
| GO | 3 | 68.82 | 56.20 | 63.94 |
| GO | 4 | 50.85 | 48.65 | 45.17 |
| GO | 5 | 195.44 | 182.19 | 211.47 |
| GO | 6 | 289.55 | 282.20 | 302.20 |
| GO | 7 | 289.19 | 274.02 | 279.00 |
| GO | 8 | 223.19 | 181.45 | 256.59 |
| GO | 9 | 801.81 | 758.78 | 670.17 |
| GO | 10 | 245.70 | 235.30 | 243.68 |
| Non-GO | 11 | 173.16 | 199.51 | 217.40 |
| Non-GO | 12 | 168.00 | 159.41 | 152.47 |
| Non-GO | 13 | 159.48 | 150.15 | 148.15 |
| Non-GO | 14 | 194.38 | 191.51 | 195.01 |
| Non-GO | 15 | 437.64 | 529.90 | 375.84 |
| Non-GO | 16 | 532.35 | 356.18 | 310.92 |
| Non-GO | 17 | 337.38 | 268.10 | 299.09 |
| Non-GO | 18 | 159.32 | 168.46 | 141.34 |
| Non-GO | 19 | 235.80 | 242.78 | 290.68 |
| Non-GO | 20 | 313.98 | 315.01 | 291.41 |

Note: Values represent the mean DAPI-normalized ORO values of three technical replicates for each donor and treatment condition before normalization to the corresponding untreated control. GO, Graves’ orbitopathy; ORO, Oil Red O; DAPI, 4′,6-diamidino-2-phenylindole.
